# Noncanonical WNT Activation in Human Right Ventricular Heart Failure

**DOI:** 10.3389/fcvm.2020.582407

**Published:** 2020-10-07

**Authors:** Jonathan J. Edwards, Jeffrey Brandimarto, Dong-Qing Hu, Sunhye Jeong, Nora Yucel, Li Li, Kenneth C. Bedi, Shogo Wada, Danielle Murashige, Hyun Tae V. Hwang, Mingming Zhao, Kenneth B. Margulies, Daniel Bernstein, Sushma Reddy, Zoltan Arany

**Affiliations:** ^1^Division of Cardiology, Children's Hospital of Philadelphia, Philadelphia, PA, United States; ^2^Cardiovascular Institute, Perelman School of Medicine, University of Pennsylvania, Philadelphia, PA, United States; ^3^Division of Cardiology, Lucile Packard Children's Hospital, Stanford University, Palo Alto, CA, United States

**Keywords:** right ventricle (RV), right ventricular (RV) failure, heart failure, Wnt, calpain, Ror2, remodeling

## Abstract

**Background:** No medical therapies exist to treat right ventricular (RV) remodeling and RV failure (RVF), in large part because molecular pathways that are specifically activated in pathologic human RV remodeling remain poorly defined. Murine models have suggested involvement of Wnt signaling, but this has not been well-defined in human RVF.

**Methods:** Using a candidate gene approach, we sought to identify genes specifically expressed in human pathologic RV remodeling by assessing the expression of 28 WNT-related genes in the RVs of three groups: explanted nonfailing donors (NF, *n* = 29), explanted dilated and ischemic cardiomyopathy, obtained at the time of cardiac transplantation, either with preserved RV function (pRV, *n* = 78) or with RVF (*n* = 35).

**Results:** We identified the noncanonical WNT receptor ROR2 as transcriptionally strongly upregulated in RVF compared to pRV and NF (Benjamini-Hochberg adjusted *P* < 0.05). ROR2 protein expression correlated linearly to mRNA expression (*R*^2^ = 0.41, *P* = 8.1 × 10^−18^) among all RVs, and to higher right atrial to pulmonary capillary wedge ratio in RVF (*R*^2^ = 0.40*, P* = 3.0 × 10^−5^). Utilizing Masson's trichrome and ROR2 immunohistochemistry, we identified preferential ROR2 protein expression in fibrotic regions by both cardiomyocytes and noncardiomyocytes. We compared RVF with high and low ROR2 expression, and found that high ROR2 expression was associated with increased expression of the WNT5A/ROR2/Ca^2+^ responsive protease calpain-μ, cleavage of its target FLNA, and FLNA phosphorylation, another marker of activation downstream of ROR2. ROR2 protein expression as a continuous variable, correlated strongly to expression of calpain-μ (*R*^2^ = 0.25), total FLNA (*R*^2^ = 0.67), calpain cleaved FLNA (*R*^2^ = 0.32) and FLNA phosphorylation (*R*^2^ = 0.62, *P* < 0.05 for all).

**Conclusion:** We demonstrate robust reactivation of a fetal WNT gene program, specifically its noncanonical arm, in human RVF characterized by activation of ROR2/calpain mediated cytoskeleton protein cleavage.

## Introduction

Right ventricular failure (RVF) is independently predictive of morbidity and mortality in diverse disease processes including left ventricular failure (LVF), pulmonary hypertension, and congenital heart disease (1–3). There is a large gap, however, in our understanding and management of RVF (4). Multiple studies demonstrate that standard reverse remodeling agents that unequivocally improve survival for LVF, such as ACE inhibitors and beta-blockers, rarely have impact in RVF, with sometimes even worsened outcomes (3, 5–7). Furthermore, markers of left ventricular remodeling poorly predict RV dysfunction (8). Thus, greater understanding of the fundamental mechanisms that drive RVF, different from LVF, is needed.

Evidence suggests that an RV-specific remodeling transcriptional program contributes to this disparate clinical behavior of the RV and LV (9). We chose to evaluate *WNT*-related differential gene expression in human RV remodeling for multiple reasons. Canonical Wnt signaling regulates cardiomyocyte proliferation during development, and is critical to second heart field development—which gives rise to the RV—but relatively dispensable for the first heart field—which gives rise to the LV (10, 11). In parallel, noncanonical Wnt signaling promotes developmental cardiomyocyte maturation, and knockout of downstream genes including *Scrib, Vangl2*, or *Rac1* results in second heart field structural anomalies and altered myocardial patterning and cardiomyocyte cell shape that resemble those seen in pathologic remodeling (12–14). In murine models of LVF and *in vitro* cardiomyocyte models, aberrant activation of both canonical and noncanonical Wnt signaling has been connected to cardiomyocyte hypertrophy, fibroblast proliferation and activation, activation of cytoskeleton remodeling, and activation of stress pathways (15–19). Unbiased transcriptomics analyses of RVF in mice due to pressure or volume-overload have identified *WNT* signaling as a pathway that is specifically altered in the progression from a compensated to decompensated state (20, 21). In humans, a recent study illustrated that higher *WNT5A* serum levels and myocardial expression correlated with worse RV, but not LV, systolic function and with higher likelihood of death or transplant in patients with dilated cardiomyopathy (DCM) (22). Together, these studies have suggested that embryonic or fetal Wnt expression may be reactivated in RV remodeling, akin to the well-established reactivation of fetal programs in LV remodeling. However, these and other studies demonstrating aberrant WNT signaling in RV remodeling have been limited to transcriptomics in murine models or were narrowly designed in humans such that the potential clinical role of WNT signaling in adaptive and pathologic remodeling, with associated preserved function (pRV) and RVF, respectively, remains incompletely defined (20–24). Finally, we also focused on WNT signaling because this pathway is dependent on extracellular factors and cell surface receptors, potentially facilitating prognostic or therapeutic avenues that target WNT signaling in RV remodeling.

LVF is an ideal human setting in which to characterize the gene expression signature of adaptive and pathologic RV remodeling for two reasons. LVF is the most common cause of RVF, and LVF causes a range of RV involvement—from pRV to RVF—reflecting different types of adaptive/pathologic RV remodeling or different points in time in disease progression (25, 26). In this study, we leverage a large collection of human RV tissues from explanted DCM and ischemic (ICM) hearts, stratified with either pRV or RVF, and from nonfailing (NF) hearts from human donors, in order to identify a robust reactivation of the fetal noncanonical WNT receptor ROR2, upregulation of ROR2/Ca^2+^ responsive protease calpain-μ, and increased cleavage of calpain-target cytoskeletal proteins specifically in severe RVF (27–29). We propose this pathway as a potential novel therapeutic target of pathologic RV remodeling.

## Materials and Methods

### Human Samples

Procurement of all myocardial tissue was performed using Gift-of-Life and University of Pennsylvania Institutional Review Board (approval 802781) approved protocols with informed consent provided when appropriate as previously described (30). RV and LV myocardial samples were retrospectively obtained from the Penn Human Heart Tissue Library collected from May 2005 to April 2018. DCM and ICM hearts were procured at the time of clinical heart transplantation. RV tissue was collected from the anterior mid free wall. LV tissue was collected from the anterolateral free wall, approximately midway between the mitral valve papillary muscles and the apex. The RV functional status (pRV vs. RVF) was identified using pretransplant right atrial (RA) pressure and RA to pulmonary capillary wedge pressure ratio (RA:PCWP) as this has ratio is predictive of RVF in the setting of LVF following left ventricular assist device placement in previously published studies (25, 26). Four cardiomyopathy RV hemodynamic patient groups were identified based on RV hemodynamics:

DCM-RVF and ICM-RVF: RA ≥ 9 and RA:PCWP ≥ 0.63

DCM-pRV and ICM- pRV: RA ≤ 8 and RA:PCWP ≤ 0.37.

Exclusion criteria included prior ventricular assist device, retransplantation, incomplete hemodynamics, or insufficient RV tissue for analysis. Potential clinical confounders to differential gene expression were assessed using chi square analysis test for categorical variables and Mann–Whitney *U* for continuous variables with pairwise deletion for any missing data. Glomerular filtration rate was calculated using the MDRD equation. All reported *P*-values were adjusted using the Benjamini–Hochberg multiple comparison correction method (adjusted *P* < 0.05 for significance).

Disease groups were compared to a NF cohort, selected from unused Gift-of-Life donor hearts that were deemed unsuitable for transplantation as previously described (30). We enriched for normal RV function by selecting those with confirmed minimal tricuspid insufficiency, preserved left ventricular function by ejection fraction ≥ 50%, and preserved renal function by creatinine ≤ 1.2.

### RNA Expression

Total RNA was extracted from coded frozen human tissue samples using RNeasy Mini Kit (Qiagen Hilden, Germany). RNA samples were diluted to 100 ng/uL by DEPC water using Qubit fluorometer (Thermo Fisher Scientific Waltham, MA). Human cDNA was synthesized using MultiScribe Reverse Transcriptase^TM^ (Thermo Fisher Scientific). Gene expression was quantified using SYBR Green Master Mix RT-PCR. RT-PCR primers (sequences in Supplementary Table 1) were validated using primer efficiency and melt curve analyses. Log2fold changes were calculated using the housekeeping genes GAPDH and TBP. Candidate WNT-related genes were identified using literature review indicating differential expression in murine models of RVF or known biological interactions (18, 20, 21, 29, 31–41). Candidate genes were first assessed for differential expression in both DCM and ICM using Kruskal–Wallis test to compare log2fold (adjusted *P* < 0.05) between NF/pRV/RVF for both the DCM and ICM cohorts. Genes that were differentially expressed for both DCM and ICM were assessed for differential expression between pRV and RVF using Mann–Whitney test after combining DCM and ICM groups.

### Protein Expression

Genes that demonstrated statistically significant differential expression between pRV and RVF were further assessed using western blot to evaluate differential protein expression. As a preliminary analysis, four representative samples from each group were selected using the lowest and highest RA:RPCW ratio for pRV and RVF, respectively, and the lowest NPPA expression for the NF controls. Protein extraction was performed using NE-PER^TM^ kit (ThermoFisher Scientific) to generate a protein library of cytoplasmic and nuclear RV myocardial fractions. Protein concentration was determined using Pierce^TM^ BCA Protein Assay kit according to manufacturer instructions (ThermoFisher Scientific). Equal amounts (~20 μg/sample) of cytoplasmic or nuclear protein, according to predicted protein location, for CREBBP, NFATC2, and ROR2 from each sample was separated by SDS-PAGE. Western blot was performed using monoclonal antibodies to the following targets: CREBBP (Cell Signaling Technology (CST), Danvers, MA, cat D6C5), NFATC2 (Abcam, Cambridge, UK, cat ab2722), ROR2 (CST cat D3B6F), TBP (Abcam, cat ab51841), HDAC2 (Abcam, cat ab32117), and GAPDH (CST, cat D16H11). Given preliminary results demonstrating significant upregulation of ROR2, western blots were performed for all remaining samples. ROR2 expression was normalized to GAPDH for each sample. Two samples that were analyzed on the first western blot assessing hemodynamic extreme samples were loaded alongside the remaining samples to allow comparisons between blots. Finally, ROR2 protein expression was normalized to median NF expression before plotting on dot plot and linear regression.

In a subset of RVF samples with either highest (*n* = 6) or lowest (*n* = 6) ROR2 expression, we performed total protein extraction using RIPA to explore activation of downstream pathways with proteins that are expressed in both cytoplasmic and nuclear fractions. Western blot was performed using antibodies to filamin A (FLNA, CST cat 4762), serine-2152 phosphorylated FLNA (CST cat 4761), calpain-μ (CST cat 2556), phosphorylated PAK1 (CST cat 2601), spectrin (Biolegend, San Diego, CA cat D8B7), and WNT5A (R&D Systems, Minneapolis, MN, cat MAB645). To determine if RVF ROR2 expression was RV-specific, we similarly assessed ROR2, FLNA, calpain-μ, serine-2152 phosphorylated FLNA, phosphorylated PAK1, and WNT5A expression in a subset of LV tissue from: nine LVF patients (six patients with RVF and high RV expression of ROR2, two with RVF and low RV expression of ROR2, and one with pRV and low RV ROR2 expression) alongside one NF LV, one NF RV, and one RVF with high ROR2 expression. Secondary anti-mouse (CST cat 7076), anti-rabbit (CST cat 7074), and anti-rat (CST cat 7077) were used as indicated. Densitometry was assessed using SuperSignal^TM^ West Femto enhanced chemiluminescent substrate (ThermoFisher Scientific) and ImageStudio (LI-COR Biotechnology, Lincoln, Nebraska).

To qualitatively determine ROR2 expression pattern, Masson's trichrome and ROR2 immunohistochemistry was performed on formalin-fixed, paraffin embedded tissue sections from a representative patient with RVF and high ROR2 expression and a pRV patient with low ROR2 expression as a negative control. ROR2 immunohistochemistry was performed using QED anti-ROR2 antibody (cat 34045) at 1:70. Immunohistochemistry and trichrome images were captured using a Nikon Eclipse 80i light microscope (Nikon, Melville, NY).

### Statistics

All data were analyzed using RStudio version 1.1.463. Data are presented as median (interquartile range), count (%), or adjusted *R*^2^ for correlations. Pairwise deletion was performed for any missing clinical data. Statistical significance was determined using Mann Whitney U for 2-group or Kruskal–Wallis for 3-group continuous variables, and chi square was used for categorical variables. All reported *P*-values were adjusted using the Benjamini–Hochberg multiple comparison correction method (adjusted *P* < 0.05 for significance).

## Results

### Patient Characteristics

We collected RV myocardial tissue from patients undergoing clinical heart transplantation for LVF due to DCM or ICM and separated them according to the presence of pRV or RVF using preexplant hemodynamic data, as described in the methods. Median time between hemodynamic data collection and explantation was 28.5 days (interquartile range: 13–53 days). In total, we identified 47 DCM-pRV, 26 DCM-RVF, 31 ICM-pRV, and 9 ICM-RVF, representing the largest and most clinically diverse study of human RV differential gene expression in LVF to date.

We identified no clinical confounders between DCM-pRV/DCM-RVF, ICM-pRV/ICM-RVF, and combined pRV/RVF groups (Table 1 and Supplementary Table 2), including no differences in gender, age, ethnicity, body surface area, body weight, heart weight, renal function by glomerular filtration rate, diabetes, use of reverse remodeling agents collectively or individually (ACE inhibitor, angiotensin receptor blocker, or β-blocker), use of other cardioactive medications (digoxin, diuretics, calcium channel blockers, or milrinone), lipid lowering medications, thyroid medications, or pacemakers. Also, ICM-RVF hearts were no more likely to have significant right coronary artery disease (≥ 70% stenosis) or history of prior coronary intervention compared to their ICM-pRV counterparts, indicating that any observed transcriptional changes could not be attributable to dichotomous coronary involvement.

**Table 1:** Clinical and demographic characteristics of right ventricular functional status by cardiomyopathy type.

	**Dilated cardiomyopathy**			**Ischemic cardiomyopathy**		
**Variables**	**RVF** ***n* = 26**	**pRV** ***n* = 47**	**Adj *P***	**RVF** ***n* = 9**	**pRV** ***n* = 31**	**Adj *P***
**Clinical and demographic characteristics**						
Age^a^	56.5 (49, 58)	52.0 (44, 60)	0.60	60.0 (56, 62)	61.0 (55, 63)	1.0
Male	50%	72%	0.60	89%	87%	1.0
Ethnicity			1.0			1.0
Caucasian	54%	64%		89%	84%	
African American	38%	30%		0%	13%	
Other	8%	6%		11%	3%	
Heart weight (grams)^a^	471 (380, 549)	465 (421, 545)	0.87	570 (512, 625)	552 (455, 639)	1.0
Weight (kg)^a^	76 (73, 91)	80 (69, 92)	1.0	93 (84, 113)	82 (75, 94)	0.92
Body surface area (m^2^)^a^	1.90 (1.8, 2.1)	1.95 (1.8, 2.1)	1.0	2.19 (2.0, 2.4)	1.99 (1.9, 2.2)	0.92
GFR^a^	49.0 (43, 65)	67.3 (55, 82)	0.10	55.4 (42, 56)	53.5 (44, 70)	1.0
Diabetes Mellitus	32%	28%	1.0	56%	39%	1.0
Insulin	15%	15%	1.0	44%	13%	1.0
Thyroid medication	31%	6%	0.14	33%	16%	1.0
Pacer	38%	21%	0.60	11%	6%	1.0
ACE inhibitor	54%	51%	1.0	56%	58%	1.0
ARB	31%	26%	1.0	22%	16%	1.0
β-blocker	81%	94%	0.60	67%	87%	1.0
Any reverse remodeling	100%	96%	1.0	100%	94%	1.0
Calcium channel blocker	8%	6%	1.0	0%	0%	1.0
Digoxin	50%	47%	1.0	22%	45%	1.0
Diuretic	100%	87%	0.60	78%	90%	1.0
Lipid lowering	42%	49%	1.0	67%	71%	1.0
Milrinone	65%	72%	1.0	86%	71%	1.0
RCA disease	4%	0%	N/A	56%	74%	1.0
Prior CABG	0%	0%	N/A	67%	43%	1.0
Prior angioplasty	4%	0%	N/A	56%	52%	1.0
Prior stent	4%	0%	N/A	56%	52%	1.0

a *Continuous variables presented as median (interquartile range) and P-value using two-tailed Mann–Whitney U test. Categorical variables presented as percent and P-value using Chi square 2 x 2 contingency tables using pairwise deletion for any missing data. All P-values are Benjamini-Hochberg corrected, and none were <0.05. ACE inhibitor, angiotensin converting enzyme inhibitor; ARB, angiotensin receptor blocker; CABG, coronary artery bypass grafting; GFR, glomerular filtration rate; RCA, right coronary artery disease*.

The NF group was similarly well-matched across all four cardiomyopathy hemodynamic groups with respect to age, ethnicity, body surface area, and weight, although not for gender (Supplementary Table 3). Previous unbiased studies of cardiac DGE have demonstrated few gender-specific differences, and none in WNT-related gene expression, suggesting a low likelihood that gender differences would impact our findings (42).

### WNT Pathway Candidate Gene Analysis

#### mRNA Expression

**Figure 1:**
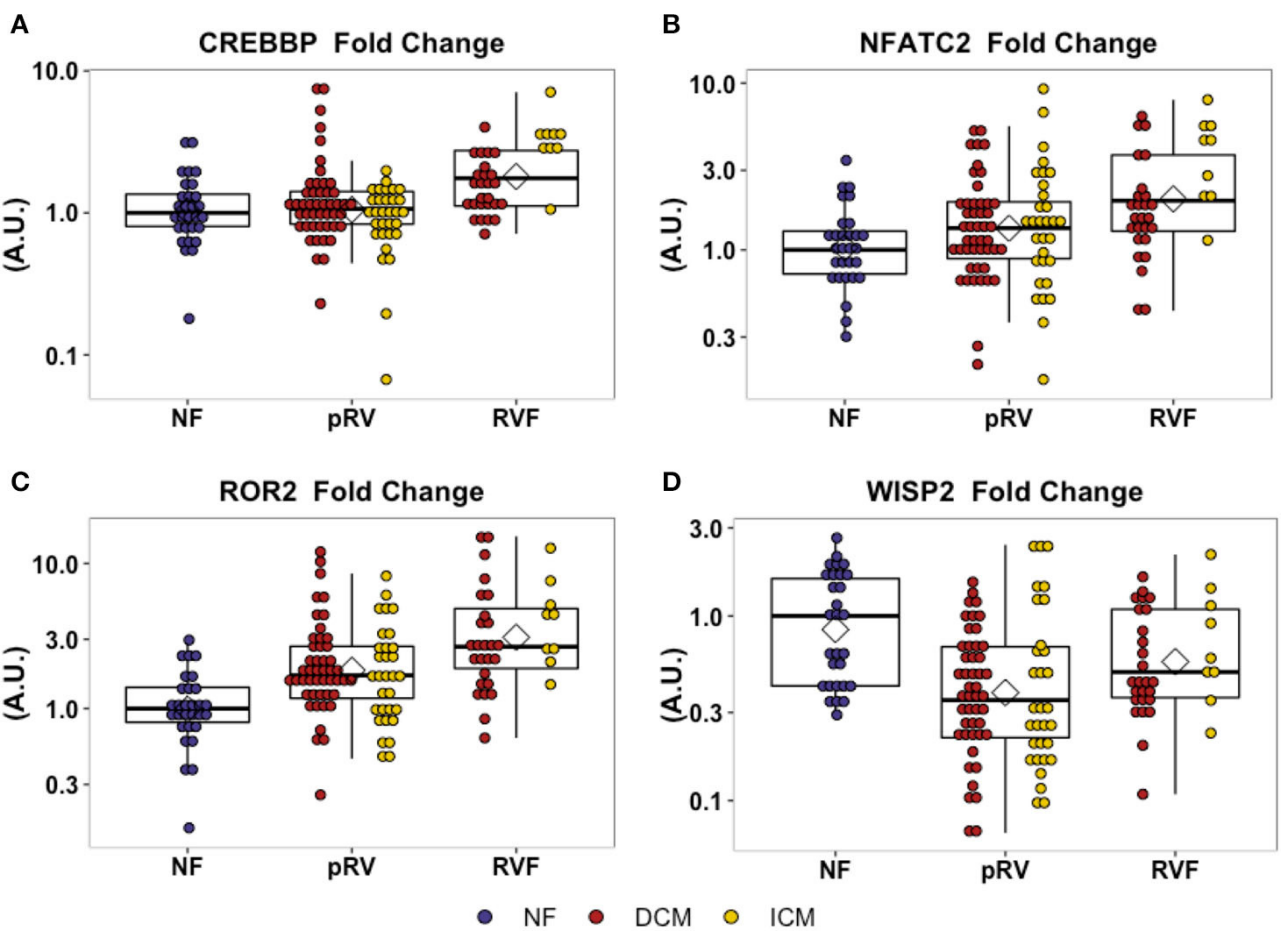
Relative mRNA expression of WNT-related genes with differential transcription between pRV and RVF as noted in **(A–D)**. WNT-related gene expression was normalized to average of GAPDH and TBP for each patient and then to the median NF expression for each gene. Genes were initially filtered for statistically significant differential expression between NF/pRV/RVF for DCM and ICM separately using Kruskal–Wallis (see Supplementary Table 4) and then were assessed for differential pRV/RVF expression using Mann–Whitney *U* (NF *n* = 29, DCM-pRV *n* = 47, DCM-RVF *n* = 26, ICM-pRV *n* = 31, ICM-RVF *n* = 9) (see also Table 2). Benjamini-Hochberg corrected *P* < 0.05 was used for significance.

In total, we assessed the RV myocardial expression of 28 WNT-related genes including ligands, receptors and co-receptors, inhibitors, and downstream signaling and transcriptional targets that have been either previously implicated in RV remodeling in murine models of RVF or have a known interaction based on literature review (Supplementary Table 1). First, we assessed whether these 28 WNT-related genes and two well-described heart failure genes—NPPA and NPPB—exhibited differential expression between NF/pRV/RVF separately for both DCM and ICM using Kruskal–Wallis to compare log_2_fold changes (43). Most WNT-related genes (Supplementary Table 4) and both natriuretic peptides demonstrated statistically significant differential expression in at least DCM or ICM, with 12 demonstrating differential expression in both: AXIN2, CREBBP, DAAM2, FZD1, FZD7, NFATC2, NPPA, ROR2, SFRP1, SFRP3, WISP2, and WNT10B. Of these 12, only five genes were found to be differentially expressed between pRV and RVF: CREBBP, NFATC2, NPPA, ROR2, and WISP2 (Table 2 and Figure 1). To further prioritize these candidate genes, we performed linear regressions comparing mRNA expression to RA:PCWP and found that ROR2 (*R*^2^ = 0.16, *P* = 3.2 x 10^−5^), CREBBP (*R*^2^ = 0.03, *P* = 0.048), and NFATC2 (*R*^2^ = 0.03, *P* = 0.048) had modest correlations with the RA:PCWP, while WISP2 did not (*R*^2^ = 0.01, *P* = 0.14).

**Table 2:** Differential mRNA Expression between RVF and pRV.

**Gene**	**RVF/pRV fold expression**	**Adj *P***
AXIN2	1.30	0.07
**CREBBP**	**1.64**	**2.63 × 10** ^**–4**^
DAAM2	1.15	0.46
FZD1	1.10	0.55
FZD7	1.19	0.46
**NFATC2**	**1.46**	**0.033**
**NPPA**	**2.68**	**0.037**
**ROR2**	**1.57**	**0.010**
SFRP1	0.90	0.31
SFRP3	1.23	0.13
**WISP2**	**1.42**	**0.039**
WNT10B	1.03	0.55

*Genes which were differentially expressed in DCM and ICM pRV/RVF compared to NF using Kruskal–Wallis were selected for further analysis comparing RVF to pRV expression using Benjamini Hochberg corrected Mann–Whitney U test (P < 0.05, bolded for significance)*.

#### Protein Expression

To assess whether protein expression would correlate with the observed transcriptional upregulation of CREBBP, NFATC2, and ROR2 in RVF, we first performed a preliminary analysis comparing four representative samples from each group representing the hemodynamic extremes for pRV and RVF and the lowest NPPA expression for NF. In this preliminary analysis, strong ROR2 protein expression was observed in the DCM-RVF samples with minimal expression in the other groups (Supplementary Figure 1). A similarly dramatic relationship was not observed for the other targets; we thus prioritized ROR2 for protein expression analysis in the remaining 123 samples.

**Figure 2:**
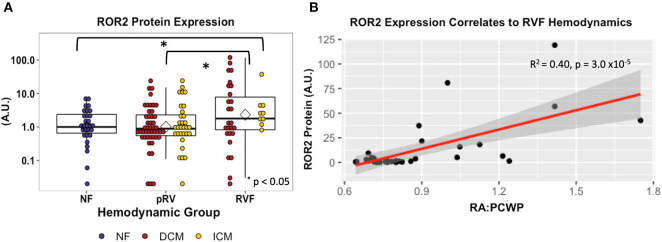
ROR2 protein expression increases in RVF and correlates with hemodynamics. **(A)** Dot plot demonstrating ROR2 protein expression (NF *n* = 29, pRV *n* = 78, RVF *n* = 35) normalized to GAPDH. To allow interblot comparison for 143 samples, two samples from the original blot (Supplementary Figure 1) were loaded alongside remaining samples on subsequent blots. Finally, expression was normalized to the median NF expression and plotted using a logarithmic base-10 scale to facilitate visual interpretation. Differential protein expression was assessed by Kruskal–Wallis comparing NF/pRV/RVF and by Mann–Whitney *U* comparing pRV/RVF. **(B)** Linear regression comparing ROR2 protein expression to RA:PCWP in RVF (*n* = 35).

ROR2 protein expression in all 143 samples correlated strongly with mRNA expression (*R*^2^ = 0.41, *P* = 8.1 × 10^−18^). Median and average RVF-to-pRV fold increase in protein expression were 2.0 and 4.5, respectively, (*P* < 0.05, Figure 2). Furthermore, particularly within the RVF group, ROR2 protein expression increased linearly with higher RA:PCWP (*R*^2^ = 0.40, *P* = 3.0 x 10^−5^, Figure 2). Finally, by using the NF ROR2 mRNA and protein expression to establish normative ranges, RVF samples demonstrated a greater than 3-fold odds (95th percentile confidence intervals) of expressing ROR2 above the 95th percentile compared to pRV (protein: OR 3.07 (1.1–8.4), *P* = 0.03; mRNA: OR 3.18 (1.4–7.3), *P* = 0.0071). In summary, ROR2 expression, both mRNA and protein, correlates directly with RVF categorically, and with worse RV hemodynamics.

**Figure 3:**
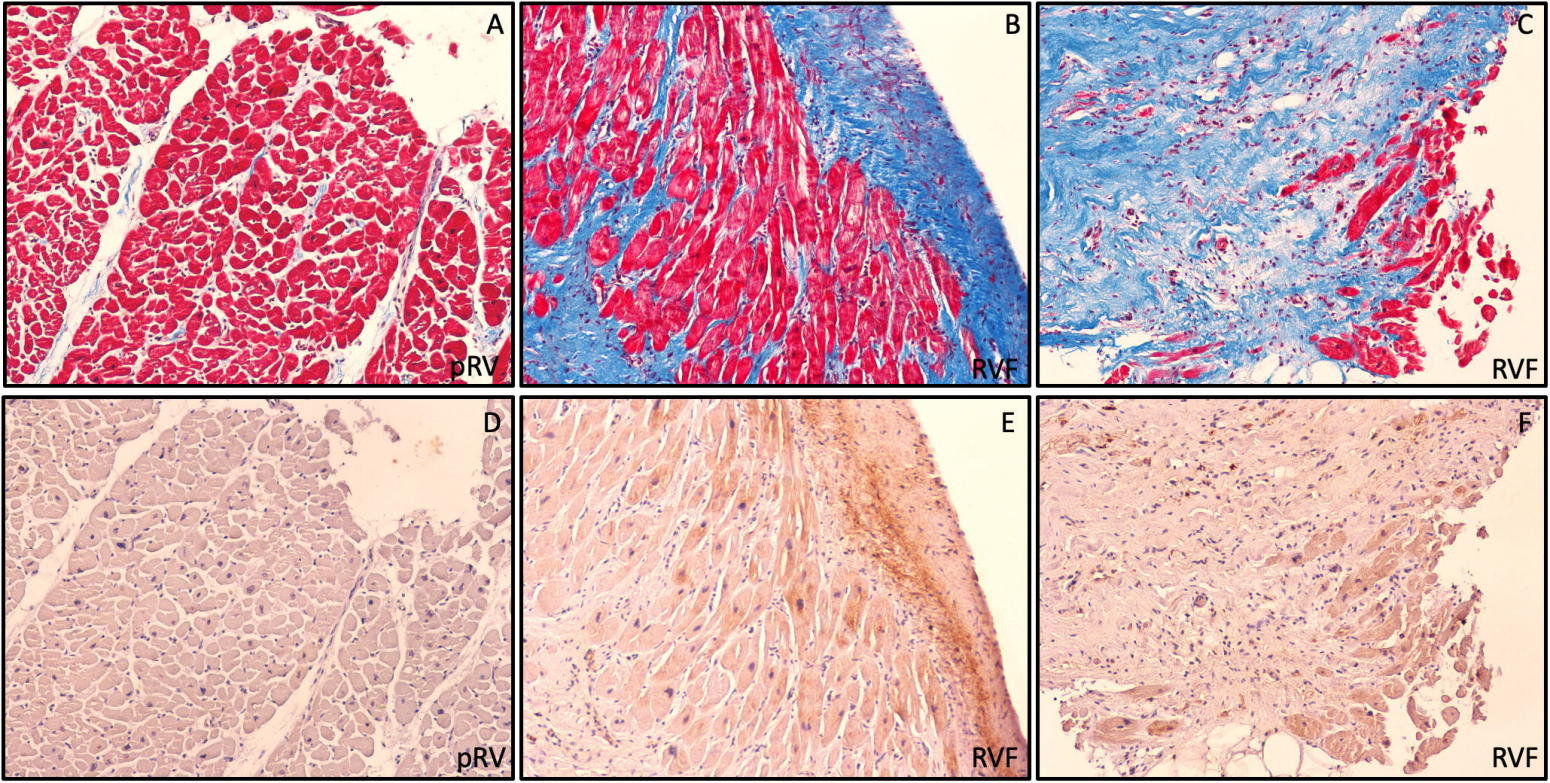
Preferential ROR2 protein expression in fibrotic areas. Representative pRV **(A,D)** and RVF **(B,C,E,F)** with low and high ROR2 expression by western blot, respectively. In these serial trichrome stained **(A,C)** and ROR2 immunohistochemistry **(D,F)** sections obtained at 10X using light microscopy, we found a pattern of preferential ROR2 expression in regions of greater fibrosis by both cardiomyocytes and noncardiomyocytes.

To qualitatively evaluate the pattern of ROR2 expression we performed Masson's trichrome staining and ROR2 immunohistochemistry from a representative RVF and pRV sample with high and low ROR2 expression, respectively. We found that ROR2 was expressed by both cardiomyocytes and noncardiomyocytes, and was preferentially expressed in areas with greater fibrosis (Figure 3).

### Impact of ROR2 Expression in RVF

#### ROR2 Expression Correlates With Actin Cytoskeletal Remodeling Pathways

**Figure 4:**
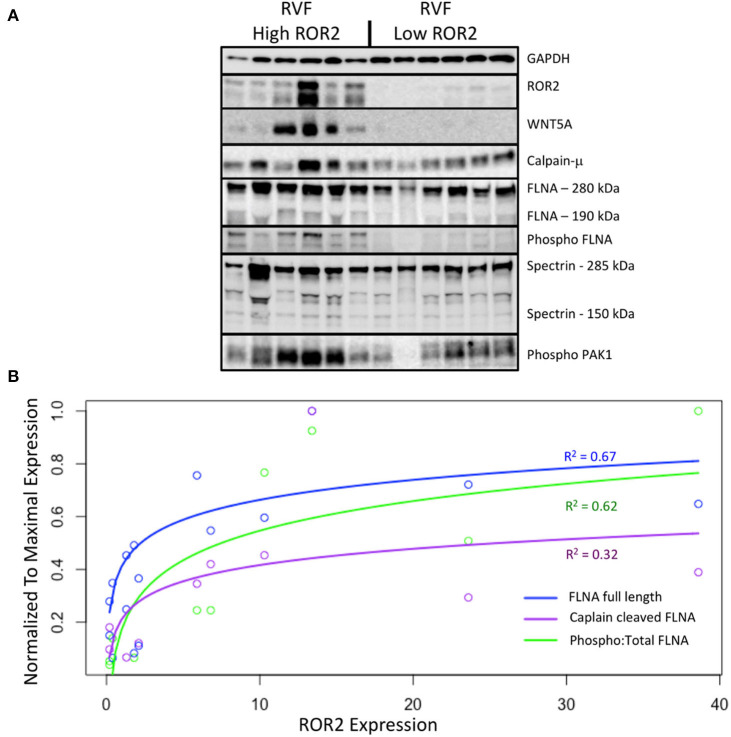
ROR2 expression correlates with increases in calpain expression and calpain-mediated cleavage. **(A)** Western blots comparing expression of downstream targets between a subset of high (*n* = 6) and low (*n* = 6) ROR2 expressing RVF patients. **(B)** Logarithmic regressions comparing ROR2 expression to full length, calpain cleaved, and phosphorylated FLNA (*n* = 12 total). Y-axis is normalized to the maximal expression of each target protein among these 12 patient samples.

We next explored what functional impact higher ROR2 expression might have in the setting of RVF by assessing overall expression and phosphorylation of downstream targets, focusing on cytoskeletal remodeling pathways. We performed repeat protein extractions for a subset of RVF samples with high (*n* = 6) or low (*n* = 6) ROR2 expression using RIPA to assess proteins expressed in both cytoplasmic and nuclear fractions. ROR2 expression was consistent between RIPA and NE-PER extraction techniques (linear *R*^2^ = 0.95, *P* = 4.7 × 10^−8^, *n* = 12). Since, WNT5A is the only known ligand for ROR2 we also assessed its expression and found significantly higher levels in patients with high ROR2 expression (fold change 37.6, *P* < 0.05, Figure 4 and Table 3).

**Table 3:** Comparative analysis of human RVF with high and low ROR2 expression.

**Target**	**ROR2 expression as continuous variable**			**High vs. low ROR2 expression**	
	**Relationship**	**Adjusted *R*^**2**^**	**Adj *P***	**Median Fold**	**Adj *P***
**Increased ROR2 expression correlates with calpain-mediated cleavage in right ventricular failure**					
**Calpain-μ**	**Linear**	**0.66**	**0.0018**	**1.8**	**0.018**
**FLNA 280 kDa**	**Logarithmic**	**0.67**	**0.0018**	**1.9**	**0.0076**
**FLNA 190 kDa**	**Logarithmic**	**0.32**	**0.045**	**3.9**	**0.0076**
**Phospho:Total FLNA**	**Logarithmic**	**0.62**	**0.0025**	**9.7**	**0.0087**
**Phosphorylated PAK1**	**Logarithmic**	**0.83**	**1.4 × 10** ^**−**^^4^	**2.1**	**0.011**
Spectrin 285 kDa	Logarithmic	0.17	0.12	1.4	0.0087
Spectrin 150 kDa	Logarithmic	0.052	0.47	1.3	0.31
**WNT5A**	**Linear**	**0.58**	**0.0050**	**37.6**	**0.0058**

*Comparative analysis of high (n = 6) vs. low (n = 6) ROR2 expressing RVF samples using ROR2 expression categorically (high vs. low) or as a continuous variable. Protein expression was normalized to either GAPDH, full length expression for cleavage fragments, or full length FLNA expression for phosphorylated FLNA. Differential protein expression was assessed by Mann–Whitney U comparing high and low ROR2 expression groups categorically. As a continuous variable, target expression was assessed using both logarithmic and linear models and the most significant model for each is displayed. In bold are comparisons that were statistically significant in both analyses*.

Normalized to GAPDH, we found a 1.4 to 1.9-fold increase in calpain-μ, full length FLNA, and full length spectrin protein expression in high ROR2 expressing RVF patients compared to low ROR2 expression (*P* < 0.05, for all). Additionally, we identified an almost 4-fold increase in the ratio of calpain cleaved FLNA (190 kDa) to full length FLNA. However, there was no significant increase in calpain cleaved spectrin (150 kDa). Phosphorylation of FLNA serine-2152 modulates its mechanosensitive interaction with integrin and inhibits calpain-mediated cleavage, and ROR2 overexpression in HEK293 cells was found to activate one of the known kinases for this site—p21-activated kinase (PAK1)—through a presumed indirect phosphorylation (44–46). We therefore assessed the relationship of ROR2 expression, FLNA phosphorylation, and phosphorylated PAK1 expression. In high ROR2 expressing RVF samples, we observed a nearly 10-fold increase in FLNA phosphorylation normalized to full length FLNA and a 2-fold increase in phosphorylated PAK1 normalized to GAPDH (*P* < 0.05, both).

Given these consistent relationships observed categorically for high and low ROR2 expression, we next assessed whether expression of these targets correlated linearly or logarithmically with ROR2 expression (Figure 4 and Table 3). The most statistically significant correlation observed was a logarithmic increase in phosphorylated PAK1 with increasing ROR2 expression (*R*^2^ = 0.83, *P* = 1.4 × 10^−4^). Full length, calpain cleaved, and phosphorylated FLNA also correlated significantly in a logarithmic relationship with ROR2 expression (*R*^2^ = 0.67, 0.32, and 0.62, respectively, *P* < 0.05). Calpain-μ and WNT5A expression correlated linearly with ROR2 expression (*R*^2^ = 0.66 and *P* = 0.0018; *R*^2^ = 0.58 and *P* = 0.0050). In summary, within the context of RVF, high ROR2 expression correlates with calpain-μ expression and calpain-mediated cleavage of FLNA as well as an increase in total FLNA and FLNA phosphorylation.

#### ROR2 Expression in LVF

To determine if ROR2 expression and evidence for activation of ROR2 targets calpain-μ and FLNA were RV-specific, we similarly interrogated these pathways in a subset of LVF samples (*n* = 9, Supplementary Figure 2). We found robust ROR2 expression in the LV of only one LVF patient who also had high RV expression of ROR2 (*n* = 6). No ROR2 expression was observed in the LV of LVF patients with RVF and low ROR2 expression (*n* = 2), LVF with pRV (*n* = 1), or NF LV (*n* = 1). Interestingly, for the one patient with high LV expression of ROR2 a similar pattern emerged with an associated robust WNT5A expression, FLNA phosphorylation, and FLNA cleavage. Thus, induction of ROR2 appears to be predominantly RV-specific, but when ROR2 is expressed in LVF, similar induction of FLNA cleavage and FLNA phosphorylation are observed.

## Discussion

To date, there are no evidence-supported therapies that target RVF (9). Our lack of understanding of the molecular mechanisms that regulate RV remodeling in humans, particularly with respect to adaptive compared to pathologic remodeling, remains a significant barrier toward achieving this goal. Here, we find compelling evidence that noncanonical WNT signaling, and in particular ROR2 signaling, is aberrantly activated in human RVF.

ROR2 is a cell surface receptor tyrosine kinase that transmits noncanonical WNT signaling following binding of its only known ligand—WNT5A—via planar cell polarity, WNT/Ca^2+^, and stress pathways including JNK/cJUN (47). ROR2 is broadly expressed during embryogenesis, being critical to cardiac, skeletal, and sympathetic nervous system development, but is silent in most healthy postnatal tissue (27, 28, 40). Global knockout of Ror1 reveals no apparent cardiac defects in the developing mouse, but knockout of Ror2 resulted in ventricular septal defects and knockout of both Ror2 and Ror1 led to conotruncal type congenital heart defects (27). Interestingly, upon our review of their published histology, we noted a significantly noncompacted appearance to the RV and LV myocardium only in the double knockout, which suggests Ror1/2 play a role in myocardial development similar to other noncanonical WNT genes (e.g., disorganized and noncompacted myocardium with loss of *Daam1, Scrib1*, or second heart field-specific loss of *Rac1*) (12–14). ROR2 in disease has been largely studied in the context of tumors, where WNT5A/ROR2 signaling leads to activation of calpain-mediated cleavage, cytoskeletal rearrangement for purposes of migration, and as a hypoxia-inducible factor downstream of *VHL/HIF* signaling with implications in tumor invasiveness and metastasis (29, 48, 49). To date, little is known about the role of ROR2 reactivation in cardiac pathology. In a rat left anterior descending ligation myocardial infarction model, increased protein levels of Wnt5a, Ror2, and Vangl2—one if its immediate downstream targets—were observed in the remote vital area (33). Interestingly, Ror2, but not Wnt5a, mRNA was also increased in the remote vital area suggesting a separate source for Wnt5a protein. We also found that protein expression of WNT5a and ROR2 correlated well, while WNT5A mRNA was not increased in RVF and did not correlate with ROR2 mRNA. Given that WNT5A is a secreted ligand, these data suggest a more paracrine or endocrine role. Using neonatal rat ventricular myocytes (NRVMs), other groups have found that Wnt5a stimulation of cardiomyocytes caused hypertrophy, cytoskeletal disruption, mPTP opening, and cJun/JNK activation, but any dependence on Ror2 or other known Wnt5a receptors was not tested (50). Our results are thus consistent with, and expand on, what few mechanistic data exist on the role of ROR2 in myocardial remodeling, and suggest that ROR2 induction in RVF mechanistically contributes to the pathogenesis of human RVF.

In this study, we found that RV ROR2 expression, both mRNA and protein, was significantly higher in patients with RVF compared to those with pRV or NF. Since ROR2 expression correlated linearly with RA:PCWP, induction of ROR2 expression was particularly strong for those with more severe RVF. We used a RA:PCWP of 0.63 to categorically define RVF given that RA:PCWP is one of the only consistent statistically significant hemodynamic predictors for RVF following left ventricular assist device placement in clinical studies (25, 26). The specific RA:PCWP cutoff to predict RVF has varied by study, but we chose to use a cutoff of 0.63 as it was an independent predictor of RVF in a multivariable analysis of patients in the HeartMate II pivotal clinical trial for bridge to transplant (*n* = 484) defined using the 75th percentile in that study. In practice, predicting RVF remains clinically challenging despite such hemodynamic markers, and our study suggests significant changes in RV biology might not occur until a higher RA:PCWP threshold is met. For instance, if a RA:PCWP of 0.85 were used to define RVF in this study, 69% of RVF patients would have ROR2 protein expression above the NF 95th percentile compared to 29% when using an RA:PCWP cutoff of 0.63. Notably, the hemodynamic extremes for DCM-RVF were higher than for ICM-RVF. If this higher RA:PCWP cut off were used, 2 (22%) of ICM-RVF and 11 (42%) of DCM-RVF would meet this criterion, which is comparable to the portion of RVF patients meeting the NF 95th percentile for ROR2 protein expression (11 and 35%, respectively for ICM-RVF and DCM-RVF).

**Figure 5:**
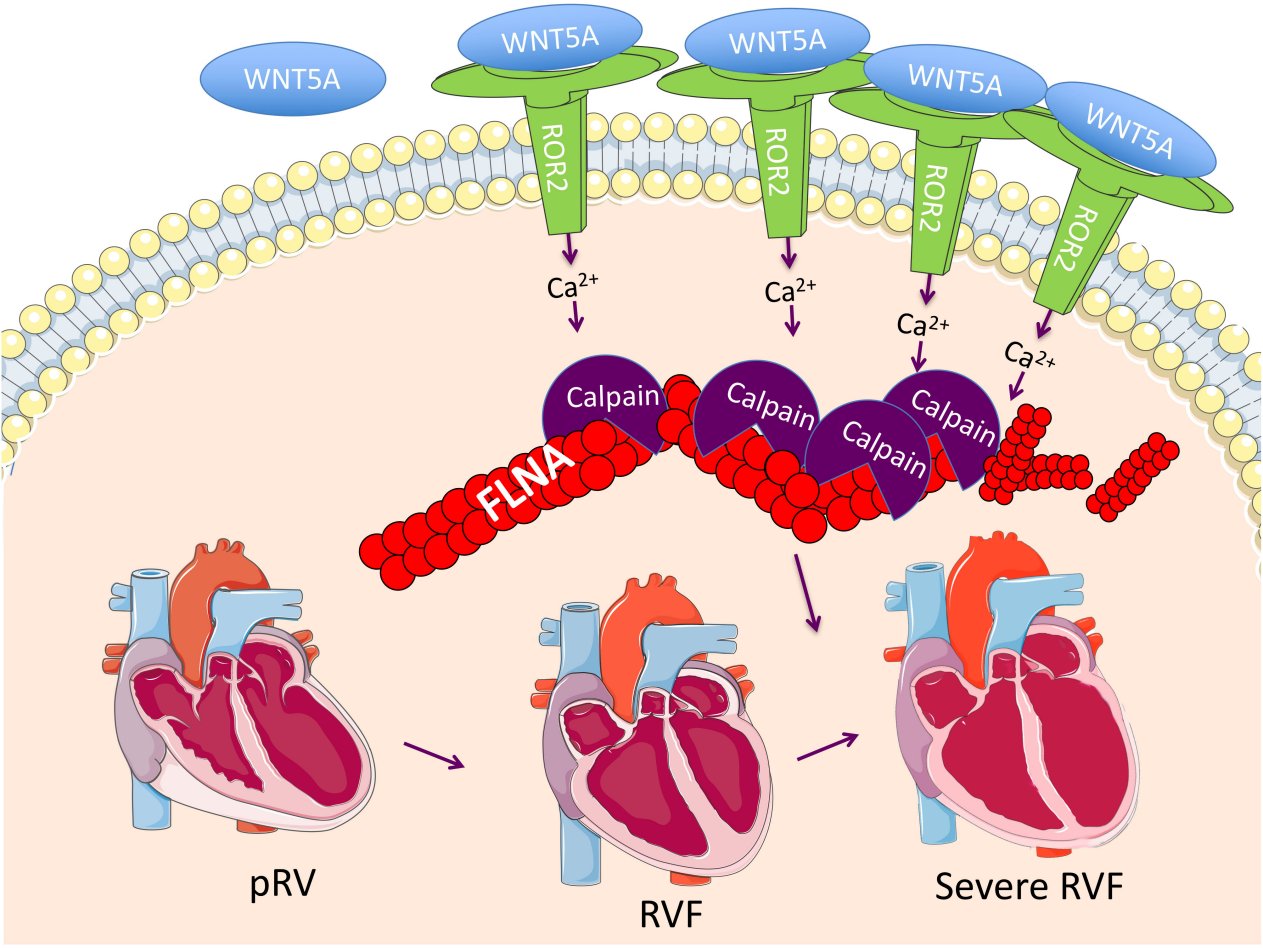
Proposed RVF model consisting of reactivation of a ROR2 fetal gene program with increasing RVF severity, which results in increased calpain expression and activity leading to calpain-mediated cleavage of cytoskeleton structural proteins including FLNA.

By comparing RVF with high and low ROR2 expression, we found evidence that in the context of RVF, ROR2 activation leads to increased calpain-mediated cleavage of FLNA. FLNA is a large scaffolding cytoskeletal protein that is involved in broad cellular functions including maintaining cell and tissue structure (e.g., cross-linking actin filaments, binding integrin, maintaining adherens junctions), cell migration, and as a signaling molecule via nuclear translocation of cleaved fragments (51). Loss of FLNA in humans or mice causes early lethality with diverse and severe developmental defects affecting the heart, lung, neurologic system, and skeleton (52). Of note, severe RV myocardial noncompaction has been previously reported in a patient with a *FLNA* G1728C mutation (53). FLNA and calpain-μ has been connected to ROR2 in cells other than cardiomyocytes. For example, in melanoma cells, ROR2-FLNA interaction is critical for WNT5A-induced JNK phosphorylation, cytoskeleton remodeling, and cell migration, and WNT5A-ROR2 binding induces calpain expression and its cleavage of FLNA leading to increased invasiveness (29, 54). Importantly, there is also evidence that calpain-μ contributes to pathologic RV remodeling. In an acute pressure overload-induced rabbit RVF model, direct right coronary artery infusion of the selective calpain inhibitor MDL-28710 partially rescued RV function and reduced cleavage of the cytoskeletal protein talin (55). Thus, our findings of ROR2 induction and activation of its downstream pathways, suggest a model (Figure 5) whereby an embryonic WNT5a/ROR2 program is reactivated in the setting of RVF, which then promotes calpain-mediated cleavage of FLNA and likely other targets, ultimately leading to maladaptive cytoskeletal changes and worsening RVF.

## Limitations

There may have been some disease progression or clinical deterioration in the time interval between tissue collection and hemodynamic assessments for DCM and ICM RV, although if so, such changes would likely have introduced variability and weakened our differential expression analyses. It is also possible that changes in gene expression occurred during the period between heart harvest and tissue freezing, although this process is highly controlled by the Penn Human Heart Tissue Library with standard operating procedures including assiduous adherence to cold cardioplegia and snap freezing samples in liquid nitrogen. Our use of rare human-derived tissue allowed us to examine differential expression in a natural setting, but it did limit our ability to mechanistically evaluate the impact of altered ROR2 expression. Thus, further work will be necessary such as testing the response to RV pressure or volume overload in Ror2 gain-of-function and loss-of-function mice.

## Conclusions

In this study, using one of the largest and most diverse existing libraries of human heart tissue, we found that WNT signaling is broadly dysregulated in RV remodeling in the setting of ICM and DCM. We found a large reactivation of embryonic ROR2 mRNA and protein expression in RVF, which correlated with worse RV hemodynamics, was observed preferentially in fibrotic regions by cardiomyocytes and noncardiomyocytes, and with activation of a downstream pathway of increased calpain expression and FLNA cleavage. Taken together, the data reveal the robust activation of noncanonical WNT signaling in human RVF, and identify ROR2 as a potential novel RVF therapeutic target that may suppress cytoskeletal remodeling.

## Data Availability

All datasets generated and analyzed for the current study are available in Supplementary Material.

## Abbreviations

CST: Cell Signaling Technology
DCM: dilated cardiomyopathy
FLNA: filamin A
ICM: ischemic cardiomyopathy
LVF: left ventricular failure
NF: nonfailing
PAK1: p-21 activated kinase
pRV: preserved right ventricular function
RA: right atrial pressure
PCWP: right atrial to pulmonary capillary wedge pressure ratio
RT-PCR: real time polymerase chain reaction
RV: right ventricle
RVF: right ventricular failure.

## References

[1] van Wolferen SA Marcus JT Boonstra A Marques KM Bronzwaer JG Spreeuwenberg MD . Prognostic value of right ventricular mass, volume, and function in idiopathic pulmonary arterial hypertension. Eur Heart J. (2007) 28:1250–7. doi: 10.1093/eurheartj/ehl477 17242010

[2] Piran S Veldtman G Siu S Webb GD Liu PP. Heart failure and ventricular dysfunction in patients with single or systemic right ventricles. Circulation. (2002) 105:1189–94. doi: 10.1161/hc1002.105182 11889012

[3] Babu-Narayan SV Uebing A Davlouros PA Kemp M Davidson S Dimopoulos K . Randomised trial of ramipril in repaired tetralogy of Fallot and pulmonary regurgitation: the appropriate study (ace inhibitors for potential prevention of the deleterious effects of pulmonary regurgitation in adults with repaired tetralogy of Fallot). Int J Cardiol. (2012) 154:299–305. doi: 10.1016/j.ijcard.2010.09.057 20970202

[4] Voelkel NF Quaife RA Leinwand LA Barst RJ McGoon MD Meldrum DR . Blood institute working group on, and f. molecular mechanisms of right heart, right ventricular function and failure: report of a national heart, lung, and blood institute working group on cellular and molecular mechanisms of right heart failure. Circulation. (2006) 114:1883–91. doi: 10.1161/CIRCULATIONAHA.106.632208 17060398

[5] Dos L Pujadas S Estruch M Mas A Ferreira-Gonzalez I Pijuan A . Eplerenone in systemic right ventricle: double blind randomized clinical trial. The evedes study. Int J Cardiol. (2013) 168:5167–73. doi: 10.1016/j.ijcard.2013.07.163 23972966

[6] Doughan AR McConnell ME Book WM. Effect of beta blockers (carvedilol or metoprolol XL) in patients with transposition of great arteries and dysfunction of the systemic right ventricle. Am J Cardiol. (2007) 99:704–6. doi: 10.1016/j.amjcard.2006.10.025 17317376

[7] Shaddy RE Boucek MM Hsu DT Boucek RJ Canter CE Mahony L . Pediatric Carvedilol Study, Carvedilol for children and adolescents with heart failure: a randomized controlled trial. JAMA. (2007) 298:1171–9. doi: 10.1001/jama.298.10.1171 17848651

[8] di Salvo TG Yang KC Brittain E Absi T Maltais S Hemnes A. Right ventricular myocardial biomarkers in human heart failure. J Card Fail. (2015) 21:398–411. doi: 10.1016/j.cardfail.2015.02.005 25725476 PMC6482959

[9] Roche SL Redington AN. Right ventricle: wrong targets? Another blow for pharmacotherapy in congenital heart diseases. Circulation. (2013) 127:314–6. doi: 10.1161/CIRCULATIONAHA.112.155887 23247301

[10] Cohen ED Wang Z Lepore JJ Lu MM Taketo MM Epstein DJ . Wnt/beta-catenin signaling promotes expansion of Isl-1-positive cardiac progenitor cells through regulation of FGF signaling. J Clin Invest. (2007) 117:1794–804. doi: 10.1172/JCI31731 17607356 PMC1891000

[11] Pahnke A Conant G Huyer LD Zhao Y Feric N Radisic M. The role of Wnt regulation in heart development, cardiac repair and disease: a tissue engineering perspective. Biochem Biophys Res Commun. (2016) 473:698–703. doi: 10.1016/j.bbrc.2015.11.060 26626076 PMC4854783

[12] Leung C Lu X Liu M Feng Q. Rac1 signaling is critical to cardiomyocyte polarity and embryonic heart development. J Am Heart Assoc. (2014) 3:e001271. doi: 10.1161/JAHA.114.001271 25315346 PMC4323834

[13] Phillips HM Rhee HJ Murdoch JN Hildreth V Peat JD Anderson RH . Disruption of planar cell polarity signaling results in congenital heart defects and cardiomyopathy attributable to early cardiomyocyte disorganization. Circ Res. (2007) 101:137–45. doi: 10.1161/CIRCRESAHA.106.142406 17556662

[14] Li D Hallett MA Zhu W Rubart M Liu Y Yang Z . Dishevelled-associated activator of morphogenesis 1 (Daam1) is required for heart morphogenesis. Development. (2011) 138:303–15. doi: 10.1242/dev.055566 21177343 PMC3005605

[15] Iyer LM Nagarajan S Woelfer M Schoger E Khadjeh S Zafiriou MP . A context-specific cardiac beta-catenin and GATA4 interaction influences TCF7L2 occupancy and remodels chromatin driving disease progression in the adult heart. Nucleic Acids Res. (2018) 46:2850–67. doi: 10.1093/nar/gky049 29394407 PMC5887416

[16] Qu J Zhou J Yi XP Dong B Zheng H Miller LM . Cardiac-specific haploinsufficiency of beta-catenin attenuates cardiac hypertrophy but enhances fetal gene expression in response to aortic constriction. J Mol Cell Cardiol. (2007) 43:319–26. doi: 10.1016/j.yjmcc.2007.06.006 17673255 PMC2084259

[17] Oerlemans MI Goumans MJ van Middelaar B Clevers H Doevendans PA Sluijter JP. Active Wnt signaling in response to cardiac injury. Basic Res Cardiol. (2010) 105:631–41. doi: 10.1007/s00395-010-0100-9 20373104 PMC2916122

[18] Barandon L Couffinhal T Ezan J Dufourcq P Costet P Alzieu P . Reduction of infarct size and prevention of cardiac rupture in transgenic mice overexpressing FrzA. Circulation. (2003) 108:2282–9. doi: 10.1161/01.CIR.0000093186.22847.4C 14581414

[19] Zuo S Jones WK Li H He Z Pasha Z Yang Y . Paracrine effect of Wnt11-overexpressing mesenchymal stem cells on ischemic injury. Stem Cells Dev. (2012) 21:598–608. doi: 10.1089/scd.2011.0071 21463175 PMC3156900

[20] Urashima T Zhao M Wagner R Fajardo G Farahani S Quertermous T . Molecular and physiological characterization of RV remodeling in a murine model of pulmonary stenosis. Am J Physiol Heart Circ Physiol. (2008) 295:H1351–68. doi: 10.1152/ajpheart.91526.2007 18586894 PMC2544484

[21] Reddy S Zhao M Hu DQ Fajardo G Katznelson E Punn R . Physiologic and molecular characterization of a murine model of right ventricular volume overload. Am J Physiol Heart Circ Physiol. (2013) 304:H1314–27. doi: 10.1152/ajpheart.00776.2012 23504182 PMC3652064

[22] Abraityte A Lunde IG Askevold ET Michelsen AE Christensen G Aukrust P . Wnt5a is associated with right ventricular dysfunction and adverse outcome in dilated cardiomyopathy. Sci Rep. (2017) 7:3490. doi: 10.1038/s41598-017-03625-9 28615692 PMC5471231

[23] Williams JL Cavus O Loccoh EC Adelman S Daugherty JC Smith SA . Defining the molecular signatures of human right heart failure. Life Sci. (2018) 196:118–26. doi: 10.1016/j.lfs.2018.01.021 29366750 PMC6310003

[24] Gaertner A Schwientek P Ellinghaus P Summer H Golz S Kassner A . Myocardial transcriptome analysis of human arrhythmogenic right ventricular cardiomyopathy. Physiol Genomics. (2012) 44:99–109. doi: 10.1152/physiolgenomics.00094.2011 22085907

[25] Kormos RL Teuteberg JJ Pagani FD Russell SD John R Miller LW . Right ventricular failure in patients with the HeartMate II continuous-flow left ventricular assist device: incidence, risk factors, and effect on outcomes. J Thorac Cardiovasc Surg. (2010) 139:1316–24. doi: 10.1016/j.jtcvs.2009.11.020 20132950

[26] Grandin EW Zamani P Mazurek JA Troutman GS Birati EY Vorovich E . Right ventricular response to pulsatile load is associated with early right heart failure and mortality after left ventricular assist device. J Heart Lung Transplant. (2017) 36:97–105. doi: 10.1016/j.healun.2016.06.015 27469015

[27] Nomi M Oishi I Kani S Suzuki H Matsuda T Yoda A . Loss of mRor1 enhances the heart and skeletal abnormalities in mRor2-deficient mice: redundant and pleiotropic functions of mRor1 and mRor2 receptor tyrosine kinases. Mol Cell Biol. (2001) 21:8329–35. doi: 10.1128/MCB.21.24.8329-8335.2001 11713269 PMC99997

[28] Morioka K Tanikawa C Ochi K Daigo Y Katagiri T Kawano H . Orphan receptor tyrosine kinase ROR2 as a potential therapeutic target for osteosarcoma. Cancer Sci. (2009) 100:1227–33. doi: 10.1111/j.1349-7006.2009.01165.x 19486338 PMC11158182

[29] O'Connell MP Fiori JL Baugher KM Indig FE French AD Camilli TC . Wnt5A activates the calpain-mediated cleavage of filamin A. J Invest Dermatol. (2009) 129:1782–9. doi: 10.1038/jid.2008.433 19177143 PMC2695838

[30] Chen CY Caporizzo MA Bedi K Vite A Bogush AI Robison P . Suppression of detyrosinated microtubules improves cardiomyocyte function in human heart failure. Nat Med. (2018) 24:1225–33. doi: 10.1038/s41591-018-0046-2 29892068 PMC6195768

[31] Burke MA Chang S Wakimoto H Gorham JM Conner DA Christodoulou DC . Molecular profiling of dilated cardiomyopathy that progresses to heart failure. JCI Insight. (2016) 1:e86898. doi: 10.1172/jci.insight.86898 27239561 PMC4882118

[32] Gomez-Arroyo J Mizuno S Szczepanek K Van Tassell B Natarajan R dos Remedios CG . Metabolic gene remodeling and mitochondrial dysfunction in failing right ventricular hypertrophy secondary to pulmonary arterial hypertension. Circ Heart Fail. (2013) 6:136–44. doi: 10.1161/CIRCHEARTFAILURE.111.966127 23152488 PMC3790960

[33] Hagenmueller M Riffel JH Bernhold E Fan J Katus HA Hardt SE. Dapper-1 is essential for Wnt5a induced cardiomyocyte hypertrophy by regulating the Wnt/PCP pathway. FEBS Lett. (2014) 588:2230–7. doi: 10.1016/j.febslet.2014.05.039 24879894

[34] Hemnes AR Brittain EL Trammell AW Fessel JP Austin ED Penner N . Evidence for right ventricular lipotoxicity in heritable pulmonary arterial hypertension. Am J Respir Crit Care Med. (2014) 189:325–34. doi: 10.1164/rccm.201306-1086OC 24274756 PMC3977729

[35] Laeremans H Hackeng TM van Zandvoort MA Thijssen VL Janssen BJ Ottenheijm HC . Blocking of frizzled signaling with a homologous peptide fragment of wnt3a/wnt5a reduces infarct expansion and prevents the development of heart failure after myocardial infarction. Circulation. (2011) 124:1626–35. doi: 10.1161/CIRCULATIONAHA.110.976969 21931076

[36] Mazzotta S Neves C Bonner RJ Bernardo AS Docherty K Hoppler S. Distinctive roles of canonical and noncanonical wnt signaling in human embryonic cardiomyocyte development. Stem Cell Reports. (2016) 7:764–76. doi: 10.1016/j.stemcr.2016.08.008 27641648 PMC5063467

[37] Nagy II Railo A Rapila R Hast T Sormunen R Tavi P . Wnt-11 signalling controls ventricular myocardium development by patterning N-cadherin and beta-catenin expression. Cardiovasc Res. (2010) 85:100–9. doi: 10.1093/cvr/cvp254 19622544

[38] Nopparat J Zhang J Lu JP Chen YH Zheng D Neufer PD . delta-Catenin, a Wnt/beta-catenin modulator, reveals inducible mutagenesis promoting cancer cell survival adaptation and metabolic reprogramming. Oncogene. (2015) 34:1542–52. doi: 10.1038/onc.2014.89 24727894 PMC4197123

[39] Sprowl-Tanio S Habowski AN Pate KT McQuade MM Wang K Edwards RA . Lactate/pyruvate transporter MCT-1 is a direct Wnt target that confers sensitivity to 3-bromopyruvate in colon cancer. Cancer Metab. (2016) 4:20. doi: 10.1186/s40170-016-0159-3 27729975 PMC5046889

[40] Takeuchi S Takeda K Oishi I Nomi M Ikeya M Itoh K . Mouse Ror2 receptor tyrosine kinase is required for the heart development and limb formation. Genes Cells. (2000) 5:71–8. doi: 10.1046/j.1365-2443.2000.00300.x 10651906

[41] Wang H Lee Y Malbon CC. PDE6 is an effector for the Wnt/Ca2+/cGMP-signalling pathway in development. Biochem Soc Trans. (2004) 32:792–6. doi: 10.1042/BST0320792 15494017

[42] InanlooRahatloo K Liang G Vo D Ebert A Nguyen I Nguyen PK. Sex-based differences in myocardial gene expression in recently deceased organ donors with no prior cardiovascular disease. PLoS ONE. (2017) 12:e0183874. doi: 10.1371/journal.pone.0183874 28850583 PMC5574577

[43] Houweling AC van Borren MM Moorman AF Christoffels VM. Expression and regulation of the atrial natriuretic factor encoding gene Nppa during development and disease. Cardiovasc Res. (2005) 67:583–93. doi: 10.1016/j.cardiores.2005.06.013 16002056

[44] Chen HS Kolahi KS Mofrad MR. Phosphorylation facilitates the integrin binding of filamin under force. Biophys J. (2009) 97:3095–104. doi: 10.1016/j.bpj.2009.08.059 20006946 PMC2793350

[45] Chen M Stracher A. In situ phosphorylation of platelet actin-binding protein by cAMP-dependent protein kinase stabilizes it against proteolysis by calpain. J Biol Chem. (1989) 264:14282–9. 2547793

[46] Goh KY Ng NW Hagen T Inoue T. p21-activated kinase interacts with Wnt signaling to regulate tissue polarity and gene expression. Proc Natl Acad Sci USA. (2012) 109:15853–8. doi: 10.1073/pnas.1120795109 23019370 PMC3465426

[47] Stricker S Rauschenberger V Schambony A. ROR-family receptor tyrosine kinases. Curr Top Dev Biol. (2017) 123:105–42. doi: 10.1016/bs.ctdb.2016.09.003 28236965

[48] Wright TM Rathmell WK. Identification of Ror2 as a hypoxia-inducible factor target in von Hippel-Lindau-associated renal cell carcinoma. J Biol Chem. (2010) 285:12916–24. doi: 10.1074/jbc.M109.073924 20185829 PMC2857057

[49] Yang CM Ji S Li Y Fu LY Jiang T Meng FD. Ror2, a developmentally regulated kinase, is associated with tumor growth, apoptosis, migration, and invasion in renal cell carcinoma. Oncol Res. (2017) 25:195–205. doi: 10.3727/096504016X14732772150424 28277191 PMC7840799

[50] Zhang P Hu C Li Y Wang Y Gao L Lu K . Vangl2 is essential for myocardial remodeling activated by Wnt/JNK signaling. Exp Cell Res. (2018) 365:33–45. doi: 10.1016/j.yexcr.2018.02.012 29454802

[51] Yue J Huhn S Shen Z. Complex roles of filamin-A mediated cytoskeleton network in cancer progression. Cell Biosci. (2013) 3:7. doi: 10.1186/2045-3701-3-7 23388158 PMC3573937

[52] Feng Y Chen MH Moskowitz IP Mendonza AM Vidali L Nakamura F . Filamin A (FLNA) is required for cell-cell contact in vascular development and cardiac morphogenesis. Proc Natl Acad Sci USA. (2006) 103:19836–41. doi: 10.1073/pnas.0609628104 17172441 PMC1702530

[53] Zenker M Nahrlich L Sticht H Reis A Horn D. Genotype-epigenotype-phenotype correlations in females with frontometaphyseal dysplasia. Am J Med Genet A. (2006) 140:1069–73. doi: 10.1002/ajmg.a.31213 16596676

[54] Nomachi A Nishita M Inaba D Enomoto M Hamasaki M Minami Y. Receptor tyrosine kinase Ror2 mediates Wnt5a-induced polarized cell migration by activating c-Jun N-terminal kinase via actin-binding protein filamin A. J Biol Chem. (2008) 283:27973–81. doi: 10.1074/jbc.M802325200 18667433

[55] Ahmad HA Lu L Ye S Schwartz GG Greyson CR. Calpain inhibition preserves talin and attenuates right heart failure in acute pulmonary hypertension. Am J Respir Cell Mol Biol. (2012) 47:379–86. doi: 10.1165/rcmb.2011-0286OC 22582173 PMC3488694

