# Supplementary material for: Noncanonical WNT Activation in Human Right Ventricular Heart Failure
Source: Front Cardiovasc Med. 2020 Oct 7;7:582407. doi: 10.3389/fcvm.2020.582407 (PMC7575695; doi:10.3389/fcvm.2020.582407)
Supplement: Supplementary file 2 [file Data_Sheet_2.docx]

**SUPPLEMENTAL MATERIAL**

**Supplemental Figure 1** – Preliminary western blot analysis of candidate genes with differential transcription between pRV and RVF

**Supplemental Figure 2** – Western blot analysis of ROR2 expression and activation in LVF

**Supplemental Table 1** – RT-PCR primer sequences

**Supplemental Table 2** – Clinical and demographic variables of combined pRV and RVF groups

**Supplemental Table 3**– Demographic variables of NF, pRV, and RVF groups for DCM and ICM separately

**Supplemental Table 4** – Fold changes by RT-PCR of WNT-related genes in DCM-pRV, DCM-RVF, ICM-pRV, and ICM-RVF


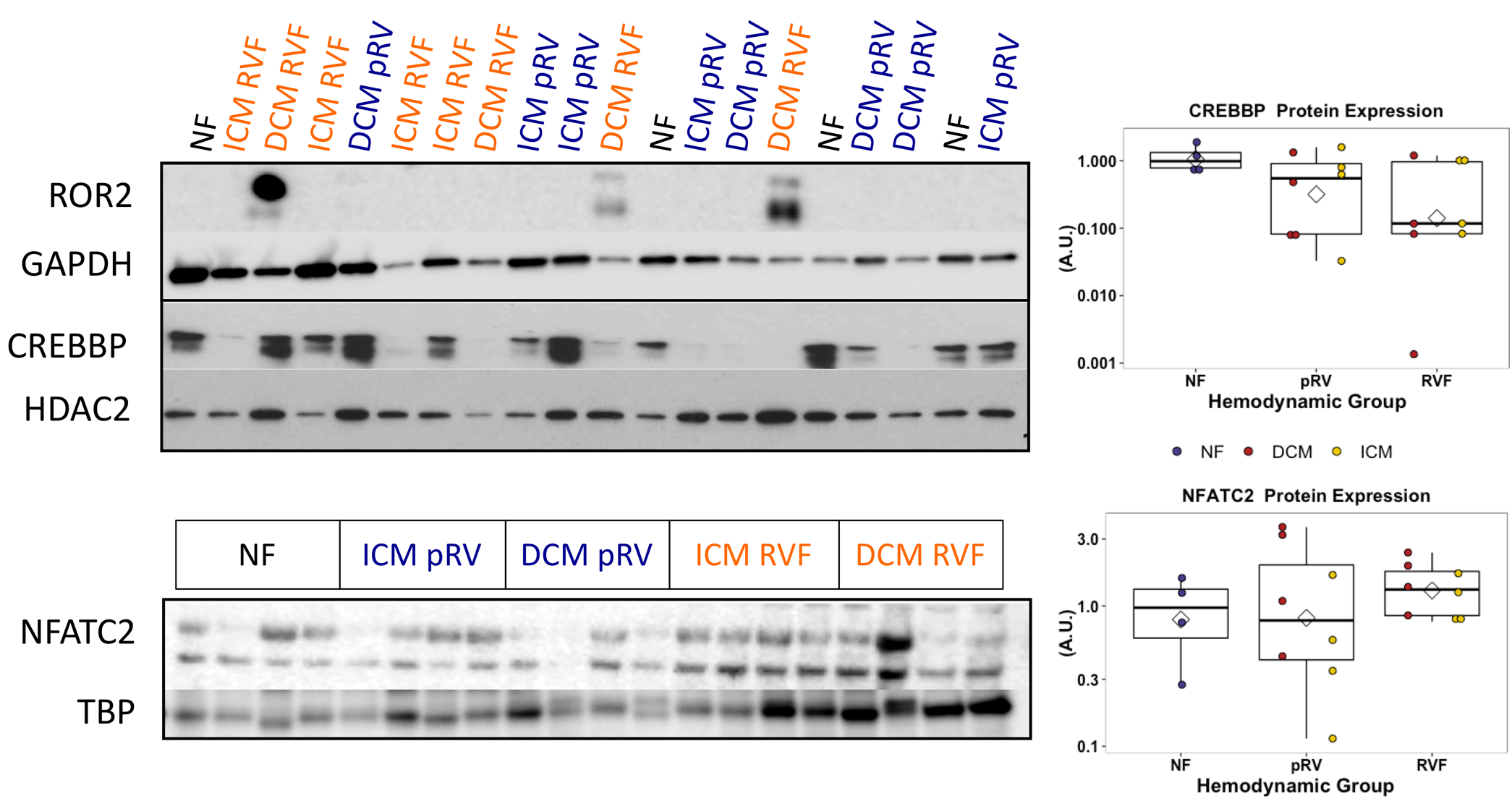


**Supplemental Figure 1**

Preliminary western blot analysis (panel A) comparing four representative samples from each group using the highest and lowest RA:PCWP for RVF and pRV, respectively, and the lowest NPPA expression for NF to represent the extremes of each group. Robust ROR2 expression was observed primarily in DCM-RVF. Dot plots (panels B, C) illustrate the relative protein expression for CREBBP and NFATC2 normalized to loading controls and to median NF expression. No statistically significant differences in expression were observed for CREBBP or NFATC2, and CREBBP protein expression trended lower in pRV and RVF samples. Robust ROR2 expression was observed primarily in DCM-RVF.


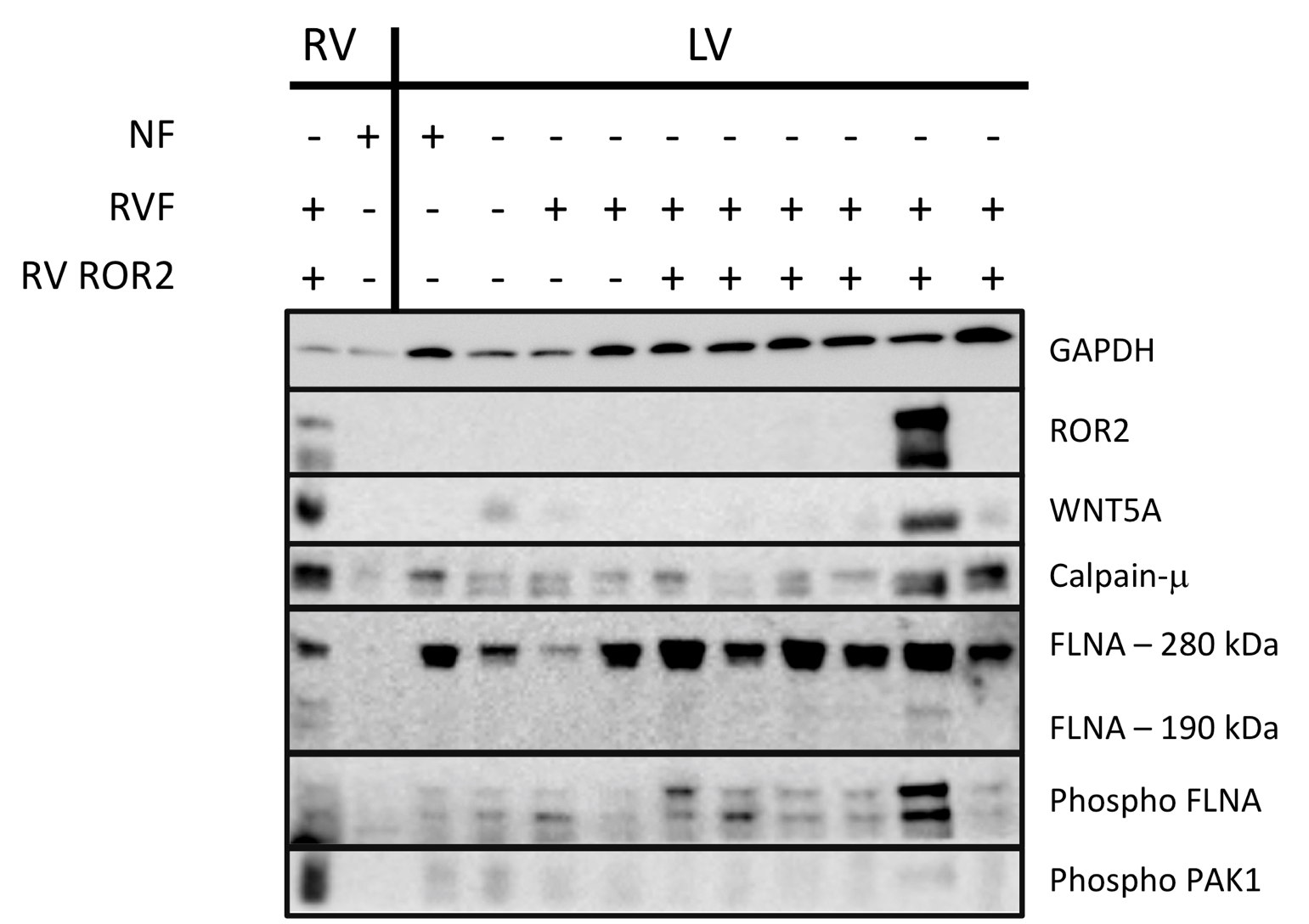


**Supplemental Figure 2**

Western blot analysis comparing RVF with high ROR2 expression (same patient as lane 4 in Figure 4), NF RV (same patient as lane 12 in supplemental figure 1), and LV tissue from patients with LVF and high RV expression of ROR2 (n = 6, same patients as lanes 1 - 6 in Figure 4) or low RV expression of ROR2 (n = 2, same patient as lanes 9 and 10 figure 4), LVF with pRV (n = 1) or NF LV (n = 1, same patient as lane 12 in supplemental figure 1). Only one LVF patient was found to have high LV ROR2 expression. Interestingly, we also observed robust expression of WNT5A, FLNA cleavage, and FLNA phosphorylation.

**Supplemental Table 1**

RT-PCR primer sequences for tested genes with references to known role in WNT signaling and/or cardiac pathology.

| **Gene Symbol** | **Forward** | **Reverse** | **Amplicon Size (bp)** | **Ref** |
| --- | --- | --- | --- | --- |
| AXIN2 | TGTCCAGCAAAACTCTGAGG | GTGCAAAGACATAGCCAGAAC | 129 | [21] |
| CAPNS1 | CACCCTGATCTGAAGACTGATG | CCTGCCACCTTTTGATGTTG | 136 | [29] |
| CREBBP | GCCGTTTACCATGAGATCCTTA | GGGTGGCAATGGAAGATGTA | 122 | [21] |
| CTNND2 | CGGCATCTCCAAAAGCAAAG | AAGACGAGGCTACAAAGTGG | 149 | [38] |
| DAAM1 | TCACCCAGAAATCACGTATCG | TCTGTGTTTGTCTGTGAGGTC | 148 | [34] |
| DAAM2 | AGATGTTTTCAGCCTACCAGAG | ATGACCGACAGCTCTTTGAC | 94 | [21] |
| DKK3 | AGGACACGCAGCACAAATTG | CCAGTCTGGTTGTTGGTTATCTT | 196 | [36] |
| FZD1 | CATCGTCATCGCCTGCTACT | TAGCGTAGCTCTTGCAGCTC | 89 | [35] |
| FZD2 | GGTGTCGGTGGCCTACAT | GAGAAGCGCTCGTTGCAC | 66 | [21] |
| FZD5 | GCACAACCACATCCACTACG | GCCATGCCGAAGAAGTAGAC | 81 | [21] |
| FZD6 | TTATGACCAGAGTATTGCCGC | AGTTTACGACAAGGTGGAACC | 150 | [21] |
| FZD7 | CGCCTCTGTTCGTCTACCTC | TCATGATGGTGCGGATACGG | 84 | [31] |
| GAPDH | GGAAGGTGAAGGTCGGAGTC | GTTGAGGTCAATGAAGGGGTC | 119 |  |
| GSK3B | CAAACAGACGCTCCCTGTG | TTCCAAAGGAATGGATATAGGCT | 83 | [21] |
| JNK1 | CCAGGACTGCAGGAACGAGT | CCACGTTTTCCTTGTAGCCC | 102 | [33] |
| NFATC2 | GAGGGGCTGTCAAAGCTCC | ACAGTTTTCCCCGTGATTCGG | 163 | [22] |
| NPPA | AGGATGGACAGGATTGGAGC | AAGTGTTGACAGGAAGCTGC | 206 |  |
| NPPB | GCTGCTTTGGGAGGAAGATG | ATGAGTCACTTCAAAGGCGG | 159 |  |
| PDE6B | AATAGGAAAGAGTGGAAGGCG | AGCAGGTTGAAGACTTGGG | 148 | [41] |
| PDK1 | CTATGAAAATGCTAGGCGTCTGT | TGGGATGGTACATAAACCACTTG | 120 | [32] |
| ROCK2 | TGATGGTTTCTATGGGCGAG | TGTTCCTACAAGTGAATCCGC | 106 | [21] |
| ROR2 | AGAATATGGTTCACGACTGCG | GTTATGATTTGGGCTGTGCG | 148 | [40] |
| SFRP1 | AAGTGTGACAAGTTCCCCG | TGGCCTCAGATTTCAACTCG | 127 | [18] |
| SFRP2 | ATGATGATGACAACGACATAATG | ATGCGCTTGAACTCTCTCTGC | 322 | [21] |
| SFRP3 | GGGACACTGTCAACCTCTATACCA | GAGCCTTCCACCAAGAGTAATCTG | 124 | [22] |
| SLC16A1 | CACCAGCGAAGTGTCATGGA | TCAAGTTGAAGGCAAGCCCA | 224 | [39] |
| WISP2 | CATAAAGACTCACAGGTCCGC | GGCAGGTACATGGTGTCG | 150 | [31] |
| WNT1 | CGGCGTTTATCTTCGCTATC | TTCGATGGAACCTTCTGAGC | 77 | [21] |
| WNT10B | GATACCCACAACCGCAATTC | GGGTCTCGCTCACAGAAGTC | 110 | [36] |
| WNT11 | GGCCAAGTTTTCCGATGCTC | CACCCCATGGCACTTACACT | 139 | [37] |
| WNT5A | GCCCAGGTTGTAATTGAAGC | TGGCACAGTTTCTTCTGTCC | 143 | [22] |

**Supplemental Table 2**

Clinical and demographic characteristics of combined DCM and ICM hemodynamic groups.

| Clinical and Demographic Characteristics | | | |
| --- | --- | --- | --- |
| Variables | RVF | pRV | Adj *P* |
|  | n = 35 | n = 78 |  |
| DCM | 74% | 60% | 0.77 |
| Age^a^ | 57  (52, 61) | 55.5  (49, 62) | 0.92 |
| Male | 60% | 78% | 0.37 |
| Ethnicity |  | | 0.88 |
| Caucasian | 63% | 72% |  |
| African American | 29% | 23% |  |
| Other | 9% | 5% |  |
| Heart weight (grams)^a^ | 499  (388, 570) | 498  (429, 609) | 0.87 |
| Weight (kg)^a^ | 84  (73, 97) | 81  (70, 93) | 0.88 |
| Body surface area (m^2^)^a^ | 1.96  (1.9, 2.2) | 1.97  (1.8, 2.2) | 0.88 |
| GFR^#^ | 50.1  (40, 62) | 62.2  (49, 81) | 0.16 |
| Diabetes Mellitus | 38% | 32% | 0.89 |
| Insulin | 23% | 16% | 0.88 |
| Thyroid medication | 31% | 10% | 0.16 |
| Pacer | 31% | 15% | 0.37 |
| ACE inhibitor | 54% | 55% | 1.0 |
| ARB | 29% | 22% | 0.88 |
| β-blocker | 77% | 92% | 0.36 |
| Any Remodeling | 100% | 96% | 0.88 |
| Calcium Channel Blocker | 6% | 4% | 1.0 |
| Digoxin | 43% | 46% | 1.0 |
| Diuretic | 94% | 90% | 0.89 |
| Lipid lowering | 49% | 59% | 0.88 |
| Milrinone | 70% | 73% | 1.0 |

^a^Continuous variables presented as median (interquartile range) and *p* value using two-tailed Mann-Whitney U test. Categorical variables presented as percent and *p* value using Chi square contingency tables using pairwise deletion for any missing data. All *p* values are Benjamini-Hochberg corrected, and none were less than 0.05. Abbreviations: angiotensin converting enzyme inhibitor (ACE inhibitor), angiotensin receptor blocker (ARB), coronary artery bypass grafting (CABG), glomerular filtration rate (GFR), and RCA (right coronary artery disease).

**Supplemental Table 3** Demographic characteristics of NF compared to RV remodeling groups.

| Demographic Characteristics Between Nonfailing and RV Remodeling Groups | | | | | | | |
| --- | --- | --- | --- | --- | --- | --- | --- |
| Variables | NF  n = 29 | DCM-pRV  n = 47 | DCM-RVF  n = 26 | Adj *P* | ICM-pRV  n = 31 | ICM-RVF  n = 9 | Adj *P* |
| Age^a^ | 56  (45, 63) | 52  (44, 60) | 56.5  (48, 58) | 0.34 | 61.0  (55, 63) | 60.0  (56, 62) | 0.56 |
| Male | **38%** | **72%** | **50%** | **0.047** | **84%** | **89%** | **4.6 x 10^-4^** |
| Ethnicity |  |  |  | 0.34 |  |  | 0.69 |
| Caucasian | 79% | 64% | 54% |  | 78% | 89% |  |
| African American | 14% | 30% | 38% |  | 13% | 0% |  |
| Other | 7% | 6% | 8% |  | 3% | 11% |  |
| Weight (kg)^a^ | 69  (62, 87) | 80  (69, 92) | 76  (73, 91) | 0.83 | 82  (75, 94) | 93  (84, 113) | 0.56 |
| Body surface area (m^2^)^a^ | 1.82  (1.7, 2.0) | 1.95  (1.8, 2.1) | 1.90  (1.8, 2.1) | 0.30 | 1.99  (1.9, 2.2) | 2.19  (2.0, 2.4) | 0.41 |

^a^Continuous variables presented as median (interquartile range) comparing NF/pRV/RVF separately for DCM and ICM using Kruskal-Wallis test. Gender and ethnicity assessed using chi-square. All comparisons corrected using Benjamini-Hochberg

**Supplemental Table 4**

Differential WNT-related gene expression in Dilated and Ischemic Cardiomyopathy Right Ventricle compared to Nonfailing

| Fold Enrichment in pRV and RVF in candidate WNT-related genes | | | | | | | | | | | | |
| --- | --- | --- | --- | --- | --- | --- | --- | --- | --- | --- | --- | --- |
|  | DCM-pRV | | DCM-RVF | |  | | ICM-pRV | | ICM-RVF | |  | |
| Gene | Fold | | Fold | | Adj *P* | | Fold | | Fold | | Adj *P* | |
| Natriuretic Peptides | | | | | | | | | | | | |
| **NPPA** | | **16.2** | | **27.9** | | **9.56 x10^-8^** | | **2.3** | | **13.1** | | **0.0023** |
| NPPB | | 9.2 | | 15.8 | | 0.026 | | 1.9 | | 4.4 | | 0.34 |
| WNT Ligands | | | | | | | | | | | | |
| WNT1 | | 2.83 | | 2.63 | | 0.0022 | | 2.09 | | 2.35 | | 0.050 |
| **WNT10B** | | **2.95** | | **2.64** | | **0.0075** | | **2.22** | | **2.58** | | **0.018** |
| WNT11 | | 1.02 | | 1.19 | | 0.89 | | 0.99 | | 2.30 | | 0.046 |
| WNT5A | | 0.84 | | 0.78 | | 0.15 | | 0.61 | | 1.11 | | 0.0025 |
| WNT receptors/co-receptors | | | | | | | | | | | | |
| **FZD1** | | **2.09** | | **1.84** | | **0.014** | | **1.69** | | **2.48** | | **0.019** |
| FZD2 | | 2.02 | | 2.05 | | 1.37 x10^-4^ | | 1.21 | | 1.42 | | 0.083 |
| FZD5 | | 0.84 | | 0.90 | | 0.34 | | 0.79 | | 1.94 | | 0.0046 |
| FZD6 | | 1.16 | | 1.19 | | 0.32 | | 1.03 | | 0.77 | | 0.73 |
| **FZD7** | | **1.81** | | **2.01** | | **4.58 x10^-7^** | | **1.85** | | **2.54** | | **3.31 x10^-6^** |
| **ROR2** | | **1.71** | | **2.57** | | **9.05 x10^-6^** | | **1.64** | | **4.32** | | **6.83 x10^-^**^4^ |
| WNT inhibitors | | | | | | | | | | | | |
| DKK3 | | 0.86 | | 0.85 | | 0.36 | | 0.87 | | 1.18 | | 0.47 |
| **SFRP1** | | **1.67** | | **1.34** | | **0.0022** | | **2.20** | | **3.01** | | **0.0012** |
| SFRP2 | | 3.04 | | 4.02 | | 0.021 | | 2.32 | | 10.08 | | 0.13 |
| **SFRP3** | | **4.26** | | **4.37** | | **1.10 x10^-10^** | | **2.76** | | **3.90** | | **2.79 x10^-^**^5^ |
| Cytoplasmic Signaling | | | | | | | | | | | | |
| **AXIN2** | | **0.62** | | **0.63** | | **0.033** | | **0.51** | | **1.18** | | **0.0013** |
| DAAM1 | | 1.11 | | 1.14 | | 0.62 | | 0.97 | | 1.02 | | 0.71 |
| **DAAM2** | | **0.52** | | **0.39** | | **0.0068** | | **0.19** | | **0.38** | | **2.79 x10^-5^** |
| GSK3B | | 1.01 | | 1.02 | | 0.94 | | 0.90 | | 1.16 | | 0.046 |
| **NFATC2** | | **1.30** | | **1.57** | | **0.032** | | **1.44** | | **4.48** | | **0.0023** |
| Target Genes | | | | | | | | | | | | |
| CAPNS1 | | 0.93 | | 1.08 | | 0.51 | | 0.99 | | 1.60 | | 0.053 |
| **CREBBP** | | **1.12** | | **1.38** | | **0.033** | | **0.95** | | **3.33** | | **6.83 x10^-^**^4^ |
| CTNND2 | | 1.19 | | 0.92 | | 0.56 | | 0.72 | | 2.35 | | 0.56 |
| JNK1 | | 1.44 | | 1.35 | | 0.0060 | | 1.31 | | 0.99 | | 0.050 |
| PDE6B | | 1.08 | | 1.10 | | 0.50 | | 0.88 | | 1.19 | | 0.15 |
| PDK1 | | 1.57 | | 1.70 | | 1.37 x10^-4^ | | 1.39 | | 1.25 | | 0.15 |
| ROCK2 | | 0.83 | | 0.80 | | 0.069 | | 0.73 | | 0.94 | | 0.023 |
| SLC16A1 | | 1.18 | | 1.08 | | 0.069 | | 0.87 | | 0.84 | | 0.99 |
| **WISP2** | | **0.39** | | **0.43** | | **8.90 x10^-4^** | | **0.31** | | **0.59** | | **0.0095** |

Differential gene expression analysis (NF normalized to 1 to assess fold change) of DCM and ICM compared to NF. Statistical significance determined using Kruskal-*Wallis* applied to log­_2_fold expression with a Benjamini-Hochberg adjusted *P* < 0.05 for significance. Genes with significant differential expression in both DCM and ICM noted in bold.
